# Orientation in Virtual Reality Does Not Fully Measure Up to the Real-World

**DOI:** 10.1038/s41598-017-18289-8

**Published:** 2017-12-22

**Authors:** Kazushige Kimura, James F. Reichert, Ashley Olson, Omid Ranjbar Pouya, Xikui Wang, Zahra Moussavi, Debbie M. Kelly

**Affiliations:** 1 Graduate Program in Biomedical Engineering, University of Manitoba, Winnipeg, Canada; 2 Department of Psychology, University of Manitoba, Winnipeg, Canada; 3 Department of Statistics, University of Manitoba, Winnipeg, Canada

## Abstract

Adult participants learned to reorient to a specific corner inside either a real or virtual rectangular room containing a distinct featural object in each corner. Participants in the virtual-reality (VR) condition experienced an immersive virtual version of the physical room using a head-mounted display (HMD) and customized manual wheelchair to provide self-movement. Following a disorientation procedure, people could reorient by using either the geometry of the room and/or the distinct features in the corners. Test trials in which the different spatial cues were manipulated revealed participants encoded features and geometry in both the real and VR rooms. However, participants in the VR room showed less facility with using geometry. Our results suggest caution must be taken when interpreting the nuances of spatial cue use in virtual environments. Reduced reliability of geometric cues in VR environments may result in greater reliance on feature cues than would normally be expected under similar real-world conditions.

## Introduction

Successful navigation requires encoding and recall of spatial cues from one’s environment. Visual-based spatial cues can be broadly categorized as either *geometric* (e.g., distance or direction) or *featural* (e.g., color or pattern). Both geometric and featural cues can be used to form a long-lasting cognitive map as first theorized by O’Keefe and Nadel in 1978^1^.

Studying how humans navigate in the real world presents researchers with a host of practical problems that can be difficult to overcome. Fortunately, advances in computer technology have enabled researchers to use realistic computer-generated virtual reality (VR) environments to simulate the real world, which provide them with some key advantages. Firstly, VR environments allow for a greater degree of control over variables of interest than real environments can typically offer^2^. Secondly, they can more easily be used in conjunction with neuroimaging techniques to study brain regions involved while a person is actively engaged in a VR navigation task^3,4^. Finally, when examining spatial memory in vulnerable populations who are physically and/or cognitively compromised^5^, VR environments offer a less stressful and physically demanding experience.

However, the advantages of using VR technology to study spatial cognition are only relevant if the processes involved in virtual navigation are similar enough to those used in real navigation that accurate inferences about spatial behavior can be drawn. Research has largely supported the effectiveness of VR in recreating the actual spatial experience, even when the VR environment is relatively non-immersive, as is the case when it is presented on a desktop display^6–11^. With the increasing availability and decreasing price point of head-mounted displays (HMD), immersive VR technology allows for physical sensations such as head movement and, when paired with external devices, locomotion. HMDs are portable and lightweight, and allow participants to obtain a greater *sense of presence* than desktop simulations can typically provide^12,13^. Overall, the use of VR as a practical method for studying spatial cognition has yielded reliable experimental results and will undoubtedly continue for the foreseeable future, especially given the continual refinement and growing accessibility of VR systems. But although many studies have used VR to examine broad questions relating to spatial cognition, few studies have specifically examined whether the nuances of how spatial cues are used in VR and real-world environments are similar.

The goal of our study was to investigate whether geometric and featural cue reliance differs between VR and real-world environments. To examine this question we employed a robust reorientation paradigm, originally used by Cheng (1986), to study spatial behavior in rats, but has since been used to study spatial behavior in a range of different animals, including humans^14–16^. The paradigm involves a rectangular room with distinct features located at each one of the corners. Typically, during a training phase, a participant learns that a reward is hidden in one of the corners (and reliably associated with one of the features), and so the participant learns to always search for the reward at that location at the exclusion of the other three corners. Using subsequent transformation tests, the spatial properties of the environment are manipulated, revealing the cues the participant was using during training (e.g., the geometric properties of the corner or the featural cue located at the corner). Using this paradigm, we were able to examine the *degree* to which participants use features and geometry in VR and real-world environments.

## Methods

### Participants

Undergraduate students (mean age = 21.7 years), with normal or corrected-to-normal vision, enrolled in the University of Manitoba’s Introductory Psychology course participated. The VR and real-world conditions had 32 participants each (16 men and 16 women). All reported experimental protocols were approved by the Psychology/Sociology Research Ethics Board at the University of Manitoba (#HS11295). The methods were carried out in accordance with these relevant guidelines and regulations. Informed consent was obtained from all participants.

### Apparatus and General Procedures: Real-World

The training and testing environment was a fully enclosed rectangular room (2.44 m wide, 4.88 m long, and 2.44 m tall) within a larger pre-existing room. The rectangular room was constructed from a wooden frame with opaque fabric curtains hung from floor to ceiling to form identical walls preventing any visual cues outside the structure from being visible. The carpeted floor was neutral-colored and contained no discernible marks or patterns. A sheet attached to the top served as a translucent ceiling and allowed the room to be uniformly lit by diffuse lighting from overhead lights. A camera (GoPro Hero 3 + Silver) was attached to the middle of the ceiling and was used to record all trials. The features (a red cube, blue cylinder, yellow cone and green sphere) all approximately fit within a size of 0.5 m wide, 0.5 m long and 0.5 m tall to maintain a relative size constancy between comparable features used in the virtual environment.

Participants were trained and tested individually. Trials began with the experimenter disorienting participants by blindfolding them and walking them randomly around the room, occasionally turning them slowly in circles, before stopping at one of four pseudo-randomized start positions located at the center of each wall. The blindfold was then removed, and participants were instructed to “find the correct corner” by approaching and pointing to their choice corner. During training trials, participants were allowed as many choices as needed to find the correct corner and received feedback after each choice; during testing trials no feedback was provided and trials ended following a single choice.

### Apparatus and General Procedures: VR Environment

**Figure 1:**
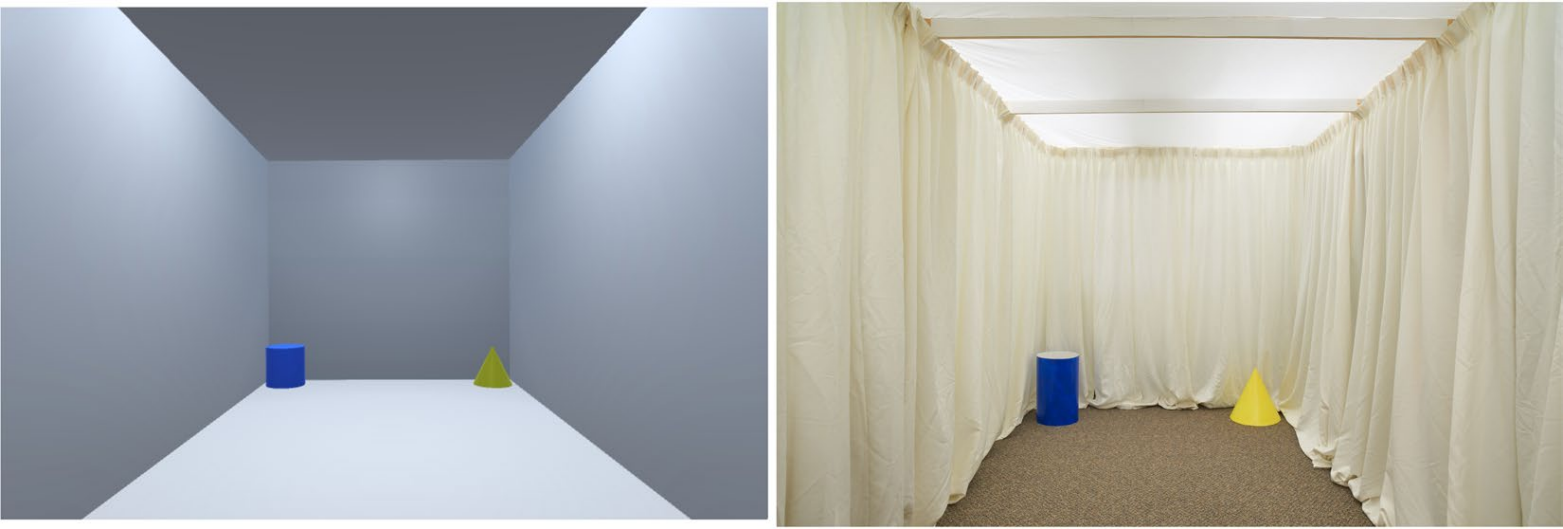
A comparison of the virtual room (left) and the real-world room (right).

The VR version of the real-world environment was measured in *virtual units* (vu) in which each vu corresponded to one meter in the real-world environment. The rectangular environment measured 3.30 vu wide × 6.87 vu long × 3.00 vu high (for a comparison of the real-world environment and virtual environment see Fig. 1). Comparable features from the real-world environment (red cube, blue cylinder, yellow cone and green sphere) were computer-rendered and located at each corner. The game engine tracked and recorded the position and orientation of the VRNChair^17^ as it moved through the VR environment.

**Figure 2:**
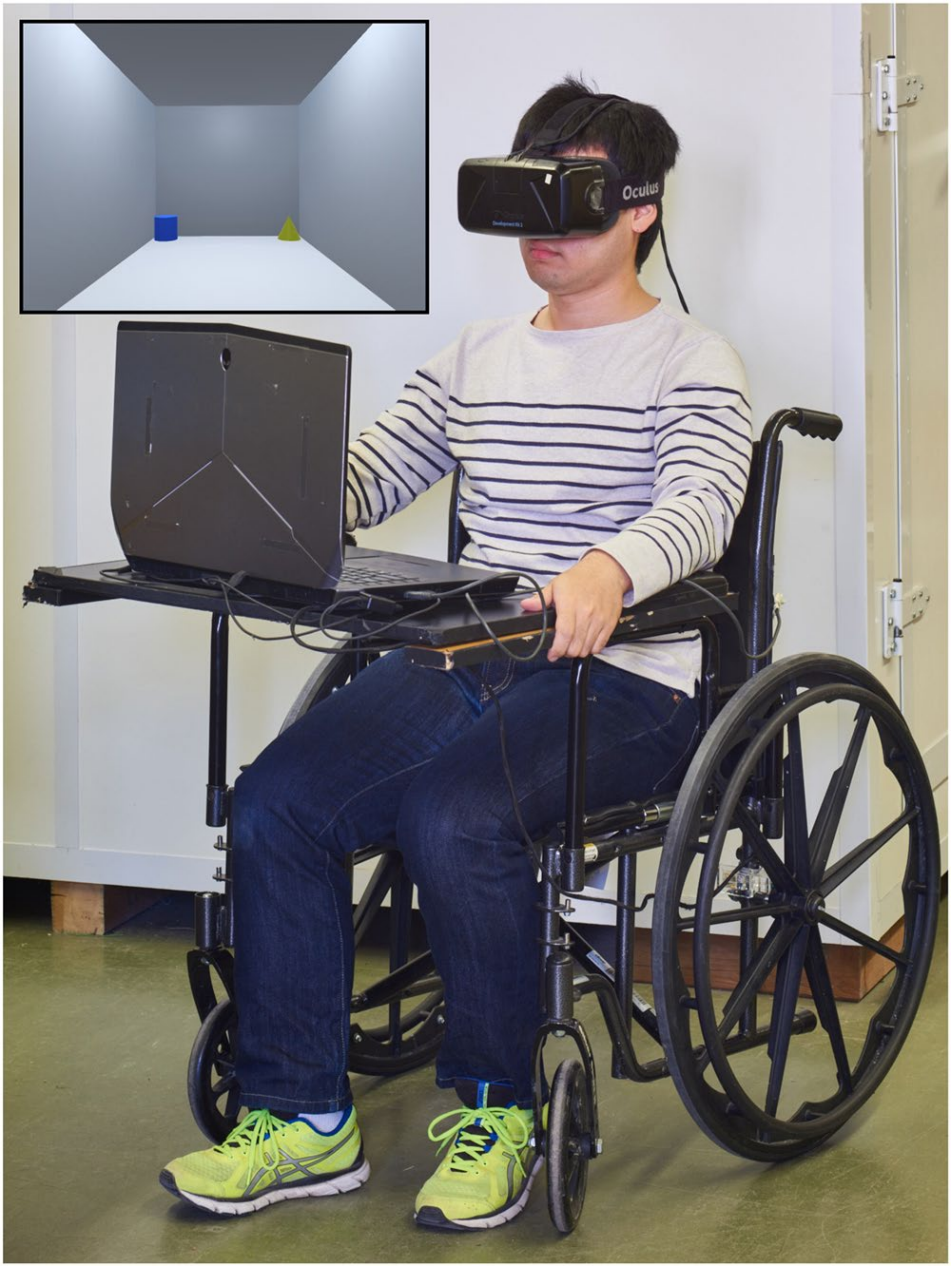
The VRNChair including laptop and HMD. The inset photo gives an example of the virtual room as viewed through the HMD. Physical movement of the VRNChair translated to movement within the virtual room. Choices were made by pressing a button attached to the participant’s right finger (not shown).

Participants were trained and tested individually. Participants viewed the virtual environment through the HMD (Oculus Rift DK2) and moved within the environment using a specially designed VRNChair (Fig. 2). The participants were instructed to “find the correct corner” and made a choice by moving to a corner and pressing a button located at their fingertip. Since the VRNChair provided an unrestricted range of movement within the VR room, participants were free to look around and fully explore the environment before making a selection. Between trials participants removed the HMD to limit any discomfort or possible vertigo. Otherwise, training and testing procedures were identical to the real-world.

### Training Phase

**Figure 3:**
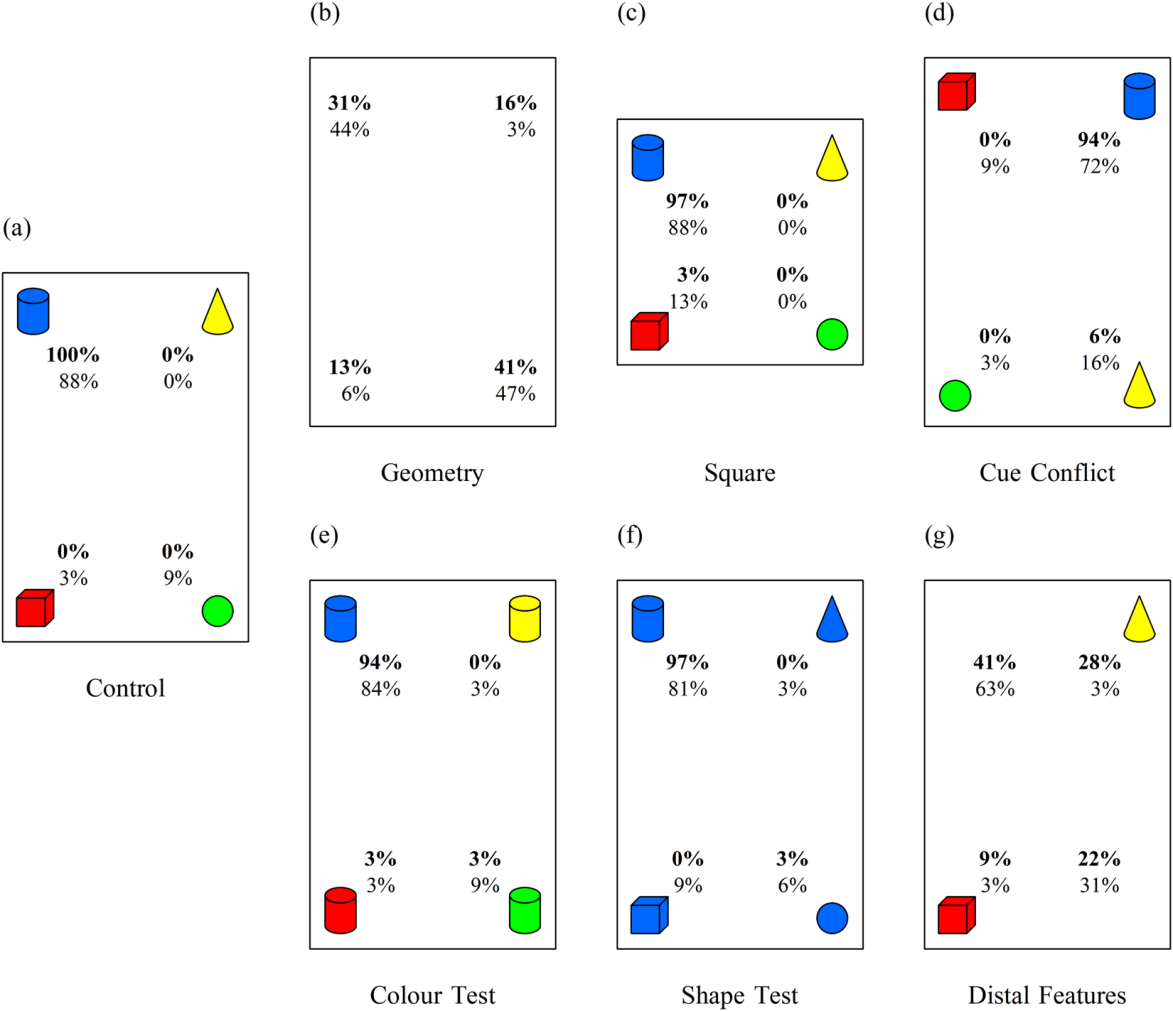
Schematic representations of the room during training/control and all testing conditions. For illustrative purposes only, the top left (i.e., the corner containing the blue cylinder) has been assigned as correct, but during the experiment this was counterbalanced across participants. Numbers represent the percentage of choices to each corner in the virtual environment and real-world environment for each testing condition (percentages for the virtual environment are indicated in bold). Note this figure is not drawn to scale.

The training phase for both the real-world and VR versions consisted of eight trials. Each participant was randomly assigned a “correct” corner, counterbalanced across participants, which was defined both by its geometry (e.g., short wall to the right of a long wall), and by the distinct feature cue located at the corner (e.g., blue cylinder; Fig. 3a). Participants were required to locate their correct corner with their first choice on the last two trials to pass training, and subsequently begin testing; participants who failed were replaced with another participant of the same sex. A total of 5 participants failed to pass training in the real-world condition and no participants failed training in the VR condition.

### Testing Phase

Six randomized testing trials, presenting a modified environment, were administered (see below). The testing phase always concluded with a training trial and a control trial (identical to the training trial except limited to a single choice and no feedback was provided). The purpose of these trials was to evaluate whether participants continued to select their correct corner after experiencing the testing phase.

#### Geometry Test

During this test, all of the features were removed from the corners, resulting in an environment void of all distinctive feature information (Fig. 3b). This test assessed whether participants incidentally encoded the geometric properties of the environment during training, despite the presence of a unique and salient feature in each of the corners (which were 100% reliable). Had the participants encoded geometry we would expect them to limit their choices to the two geometrically correct corners (the correct corner and its rotational equivalent), whereas if they had not encoded geometry we would expect an equal distribution of choices among the four corners.

#### Square Test

During this test, the features were organized in the same arrangement relative to training, but the environment itself was square in shape - all walls were equal in length thus removing any informative geometric information (Fig. 3c). This test examined whether participants could use the feature cues when all informative geometric cues had been removed, by limiting their choices to the one featurally correct corner.

#### Cue Conflict Test

During this test, the four features from training were present, but each feature was relocated one corner clockwise from its position during training (Fig. 3d). This test deliberately placed geometric and featural information in conflict by placing the correct feature in a geometrically incorrect corner. Therefore, this test required participants make a choice based on either geometric (choosing between the two geometrically correct corners) or featural information (choosing the corner which contained the correct feature), which allowed us to examine participants’ preference, or weighing, of these cues.

#### Colour Test

During this test, all four features retained their same colour property as during training, but the shape property was modified such that they were all the same shape as the feature in the participant’s correct corner from training (Fig. 3e). For example, if the feature in the correct corner had been a blue cylinder, all the other features during this test would be shaped as a cylinder (i.e., a yellow cylinder, red cylinder, blue cylinder, and green cylinder). This test examined whether participants had encoded the colour of their correct feature during training, independent of its shape.

#### Shape Test

During this test, all four features retained their same shape property as during training, but the colour property was modified such that they were all the same colour as the feature in the participant’s correct corner during training (Fig. 3f). For example, if the feature in the correct corner had been the blue cylinder, all the other features during this test would be blue (i.e., a blue cone, blue cube, blue cylinder, and blue sphere). This test examined whether participants had encoded the shape of their correct feature during training, independent of colour.

#### Distal Features Test

During this test, the feature in both the correct corner and the diagonally opposite corner were removed (Fig. 3g), leaving only the two features in the geometrically incorrect corners. This condition examined whether participants were able to use features in the distal corners to determine the position where the correct corner should be located.

### Statistical Analyses

Each testing condition contained either a correct response whereby a person received a score of 1 or an incorrect response whereby a person received a score of 0; the criteria for determining what defined a correct response differed for each testing condition. Binomial tests were used to examine the percentage of choices made to the correct corner(s) for each testing condition separately for the real-world and virtual environments. Independent proportions z-tests were then used to compare the percentage of correct responses between the real-world and virtual environments for each testing condition.

As both the Geometry and Distal Features tests allowed for a strategy whereby a choice was considered correct if it was made to either of the geometrically correct corners (i.e., the correct corner or its diagonal opposite, herein referred to as the *rotational corner*), additional Wilcoxon signed-rank tests examined whether choices differed between these two corners.

### Data Availability

All data generated or analyzed during this study are included in this published article (and its Supplementary Information files).

## Results

### Control Test

The purpose of the Control Test was to confirm participants retained knowledge of their correct corner throughout the testing phase. During this test, all features were located in the corners as during training, with the only difference being no visual or verbal feedback was provided after the participant made a choice. A choice to the correct corner was considered correct and a choice to any other corner was considered incorrect.

Binomial tests showed the percentage of choices to the correct corner significantly exceeded choices to all other corners in both the real-world (91% vs. 9%, respectively; *p* < 0.001) and VR environment (100% vs. 0%, respectively; *p* < 0.001). A comparison of correct choices did not differ between the real-world and VR environments (Z = 1.77, *p* = 0.08).

Overall, results from the Control test show that participants in both environments retained a memory for their correct corner throughout the training phase and in the absence of feedback (Fig. 3a).

### Geometry Test

The purpose of the Geometry test was to determine whether participants had incidentally encoded the geometric properties of the room during training. During this test, all features were removed from the corners and only the geometric cues from the properties of the environment itself were informative. Since the correct corner and the rotational corner were geometrically identical, a choice to either of these corners was considered a correct choice and a choice to either of the two remaining corners was considered an incorrect choice.

Binomial tests showed the percentage of choices to the correct geometric corners significantly exceeded choices to the incorrect geometric corners in both the real-world (91% vs. 9%, respectively; *p* < 0.001) and VR environment (72% vs. 28%, respectively; *p* = 0.02). Additional Wilcoxon signed-rank tests showed choices to the positive and rotational corners did not differ in either the real-world (Z = 0.186, *p* = 0.853) or the VR environments (Z = 0.626, *p* = 0.532), indicating participants were unable to distinguish between these two geometrically identical corners (this is strong evidence the participants were not using any uncontrolled cues, such as hallway noise in the real-world room). A comparison of geometrically correct choices between the real-world and VR environments was marginally significant (Z = 1.92, *p* = 0.052).

Together these results show that geometry was encoded incidentally in both environments as participants were able to accurately use the geometric properties in both the real-world and VR settings, although geometric encoding was more pronounced in the real-world (Fig. 3b).

### Square Test

The goal of the Square Test was to determine whether participants had encoded the feature in their correct corner, and could use this cue independent of geometry. During this test any informative geometric cues were removed leaving the four distinctive features from training. A correct choice was one made to the corner containing the correct feature and an incorrect choice was one made to any of the other three corners.

Binomial tests showed the percentage of choices to the corner containing the correct feature significantly exceeded choices to all other corners in both the real-world (88% vs. 12%, respectively; *p* < 0.001) and VR environments (97% vs. 3%, respectively; *p* < 0.001). A comparison of choices to the correct feature between the real-world and VR environments was not significant (Z = −1.4, *p* = 0.16).

Overall, results from the Square test showed participants could use their correct feature to reorient independent of informative geometry in both the real-world and VR environment (Fig. 3c).

### Cue Conflict Test

The purpose of the Cue Conflict test was to determine whether participants relied more on either featural or geometric cues when the two cue types provided conflicting information as to the location of the correct corner. During this test, each feature was repositioned one corner clockwise from its location during training, meaning if a participant made a correct featural choice they would do so by making an incorrect geometric choice (and vice-versa).

Binomial tests showed the percentage of choices to the correct feature significantly exceeded choices to the correct geometry in both the real-world (72% vs. 28%, respectively; *p* = 0.02) and VR environments (94% vs. 6%, respectively; *p* < 0.001). A comparison of featurally correct choices between the real-world and VR environments was significant (Z = −2.32, *p* = 0.02), indicating a stronger preference for choosing the correct location according to featural information in the VR environment compared to the real-world environment.

Overall, results from the Conflict test showed participants relied more heavily upon featural cues over geometric cues when these two sources provided conflicting information as to the goal location. Furthermore, this weighing of featural cues was heavier for participants in the VR environment than for participants in the real-world environment (Fig. 3d).

### Colour Test

The purpose of the Colour test was to determine whether participants had encoded the colour of their correct feature. During this test, the colour of each feature was different (and consistent with training) but all were the same shape as the correct feature. A choice was considered correct if it was made to the corner containing the feature with the same colour as the correct feature during training; a choice was incorrect if it was made to any of the other three corners.

Binomial tests showed the percentage of choices to the corner containing the correct-coloured feature significantly exceeded choices to all other corners in both the real-world (84% vs. 16%, respectively; p < 0.001) and VR environments (94% vs. 6%, respectively; p < 0.001). A comparison of correct choices between the real-world and VR environments was not significant (Z = −1.2, p = 0.23).

Overall the results show participants had encoded the shape of their correct feature independent of the colour in both the VR and real-world environments (Fig. 3e).

### Shape Test

The purpose of the Shape test was to determine whether participants had encoded the shape of their correct feature. During this test, the shape of each feature was different (and consistent with training) but all were the same colour as the correct feature. A choice was considered correct if it was made to the corner containing the feature with the correct shape as during training; a choice was incorrect if it was made to any other corner.

Binomial tests showed the percentage of choices to the corner containing the correct-shaped feature significantly exceeded choices to all other corners in both the real-world (81% vs. 19%, respectively; *p* = 0.001) and VR environments (97% vs. 3%, respectively; *p* < 0.001). However, unlike the Colour test, a comparison of correct choices between the real-world and VR environments was significant (Z = −2.0, *p* = 0.046), indicating a more accurate encoding of the correct feature’s shape in the VR environment compared to the real-world environment.

Overall the results show participants had encoded the shape of their correct feature when they were learning the task, and this encoding was more accurate in the VR environment than it was in the real-world environment (Fig. 3f).

### Distal Features Test

The goal of the Distal Features test was to determine whether participants had encoded the features located in the two geometrically incorrect corners (the distal corners), or whether the participants had only encoded those feature(s) in the geometrically correct corners. During this test the features in the correct corner and rotational corner were removed (geometrically correct corners) leaving only the features in the two geometrically incorrect corners; the feature in the rotational corner was removed to eliminate the possibility that participants could determine their correct corner as the only one without a feature.

Binomial tests showed the combined percentage of choices to the geometrically correct corners was significantly greater than choices to the geometrically incorrect corners in the real-world environment (94% vs. 6%, respectively; *p* < 0.001) but not in the VR environment (63% vs. 37%, respectively; *p* = 0.215). A comparison of combined choices to these corners between the real-world (94%) and VR environments (63%) was significant (Z = 3.02, *p* = 0.003), showing participants in the real-world room chose these geometrically correct corners more than participants in the VR room; indeed participants’ choices in the VR room were not significantly different from chance (50%; *t* = 1.44, *p* = 0.161; one-sample t-test).

Wilcoxon signed-rank tests comparing choices between the correct and rotational corners were not significant in either the real-world (63% vs. 31%, respectively; Z = 1.83, *p* = 0.068) or VR environment (41% vs. 22%, respectively; Z = 1.34, *p* = 0.18), establishing that participants were not able to choose the location of their correct corner more than the rotational corner in either environment type.

Overall, results show participants did not encode the relative position of the distal features in either type of environment. However, when only the distal features were available the participants in the real-world resorted to using the geometric cues, whereas the participants in the VR environment did not choose the geometrically correct corners more often than chance (Fig. 3g).

## Discussion

Overall, our results support two main conclusions. Firstly, they support the general use of VR for studying spatial navigation, although caution must be taken when interpreting how participants are encoding spatial cues. Participants were easily able to navigate the VR room, using the HMD for visual input and the customized wheelchair (VRNChair) to provide movement, and the spatial decisions they made were *generally* comparable to those in the real-world room. However, clearly the use of spatial cues differed in *degree* - geometric encoding was not as accurate in the VR environment as in the real-world environment, leading to our second main point. Although the geometry and feature properties were encoded by participants in both environments, the geometric cues were relied upon more heavily in the real environment than in the VR environment. Thus, geometry in virtual settings appears not to be encoded by participants in a manner analogous to the real-world, even in our highly immersive VR procedure. This is an important finding for all studies of orientation and navigation using a VR approach.

### Encoding Features

The main test to determine whether people had encoded feature cues independent of the room geometry was the Square test. During this test, all walls of the room (either VR or real-world) were of equal length which removed any informative geometric information, but the four features from training were still present. Not surprisingly, in both the VR and real-world rooms, participants chose the corner containing their correct feature, thus establishing they were able to use their correct feature in environments without informative geometric information. During the Cue Conflict test, which was conducted in a rectangular-shaped room, each feature was repositioned to the nearest corner clockwise to where it had been during training, a condition that placed featural and geometric information in conflict. In both the VR and real environments, participants chose the corner that contained their correct feature even though it was now in a geometrically incorrect corner, thus showing primary weighing of features in both environments.

To determine whether people had encoded the colour and shape of their correct feature independently, two additional tests were conducted whereby these two properties were dissociated. The Colour test showed people could identify the correct corner using only colour information and the Shape test showed they could identify the correct corner using only shape information; this finding was consistent in both the VR and real-world conditions. However, one unexpected finding was participants in the VR environment were more accurate at using shape information from the features compared to those in the real environment. At present we are not sure why shape information per se may have been more salient for people in the VR condition. However it should be noted that shape recognition was high in both environments (97% in VR and 81% in real-world) and these differences were only moderately different from those of colour encoding (94% in VR and 84% in real-world).

The tests described thus far support that people in both the VR and real-world environments clearly encoded their correct feature during training procedures. But did they also encode the features located in the other distal (geometrically incorrect) corners? This question was examined during the Distal Features test in which only the features located in the two geometrically *incorrect* corners were present during test trials. Had people sufficiently encoded either (or both) of these features, they could have used this information to identify their correct corner, allowing them to limit their choices to only this correct corner and avoid choosing the rotationally equivalent corner. This did not happen, either in the VR or the real environment, suggesting people relied upon the feature located in their correct corner, and failed to encode the relative location of the other features.

### Encoding Geometry

During the Geometry test, all the features present during training were removed, leaving only wall length and left-right sense to guide people to their correct corner. Results from the Geometry test in both the VR and real-world rooms showed people could use geometry to focus their choices to the geometrically correct corners (72% in VR and 91% in real-world). The conclusion therefore is people in both the VR and real-world rooms encoded the geometry of the space itself. But was the accuracy of this encoding equivalent in both environments? When comparing correct geometric choices between VR and real-world environments there was a marginally significant (*p* = 0.052) advantage favoring more accurate encoding of geometry in the real-world compared to the VR environment. This finding suggests environmental geometry may have been a more salient (and consequently more useful) spatial cue in the real-world than it was in the VR room, an interpretation that is further supported by results from both the Cue Conflict and Distal Features test.

During the Cue Conflict test the features from training were present in each of the corners, except each feature was now relocated one corner clockwise from the corner it occupied during training. This arrangement required participants make a choice between either the correct geometry or the correct feature. In both the VR and real-world rooms participants chose the corner with the correct feature over the geometrically correct corners, but this preference was significantly stronger in the VR environment than in the real-world environment (94% vs. 72%), a finding that can reasonably be attributed to weakened influence of competing geometry in the VR room. Furthermore, during the Distal Features test, although overall the participants were unable to use distal features to choose their correct corner in either environment, those in the real-world room resorted to a geometric strategy, whereas participants in the VR room did not. This result again supports geometric information was more salient in the real-world environment compared to the VR environment.

The findings of reduced sensitivity or saliency of geometry in the VR room is consistent with similar research showing that distance perception is generally underestimated in VR environments compared to real environments^18–20^. A possible explanation for this underestimation is the field of view provided by the HMD (approximately 100 degrees) is narrower than our natural visual field. However, previous experiments have shown that specifically reducing observers’ field of view does not have a direct effect on distance perception in either real-world^21^ or virtually-rendered environments^22^. A second possibility is binocular depth perception may have been compromised for people who experienced the VR condition. Binocular depth perception results from the slight displacement of the visual field between the left and right eye^23^, a condition that is simulated inside the HMD through the calibration of inter-pupil distance for each person. However, since this calibration depends on the subjective responses of the person as he/she views images only in VR, perfect calibration is often difficult to achieve since slight errors typically go unnoticed by the user^24^. Therefore, the binocular depth cues provided by HMDs in general can best be described as a close approximation of the real-world experience, with any differences being expressed in the imperfect perception of geometric properties.

## Conclusions

During this study we found that reorientation strategies in VR and real-world environments were qualitatively equivalent. People encoded the featural and geometric cues, and when required to make a choice between these two cue types they chose features, regardless of whether it was in a VR or real-world setting. But upon closer inspection, the use of these spatial cues also differed in *degree* in that geometric encoding was not as accurate in the VR environment as in the real-world environment. Given that much of the current reorientation and navigation research is conducted using virtually rendered environments^25–28^, our findings provide an important level of insight (as well as a point of caution) for researchers when interpreting results from VR settings. The metric properties of virtual environments appear not to be encoded by participants in a manner analogous to encoding in real-world environments, even in a highly immersive VR environment as was used in our current study. For VR applications designed to meet the needs of a more general public (e.g., gaming or information displays) this discrepancy is not likely to pose a problem, but for the more stringent standards required of spatial cognition research this is an issue which needs to be acknowledged and addressed.
